# Ramadan intermittent fasting and the gut microbiome: modulation of diversity and implications for metabolic health

**DOI:** 10.3389/fnut.2026.1767573

**Published:** 2026-05-28

**Authors:** Shahla Shahbazi, Soheila Aminzadeh, Majid Taati Moghadam, Sajad Rajabi, Seyedeh Shadi Vaziri, Mosayeb Rostamian

**Affiliations:** 1 Infectious Diseases Research Center, Health Policy and Promotion Institute, Kermanshah University of Medical Sciences, Kermanshah, Iran; 2 Department of Toxicology, Faculty of Pharmacy, Ahvaz Jundishapur University of Medical Sciences, Ahvaz, Iran; 3 Toxicology Research Center, Medical Basic Sciences Research Institute, Ahvaz Jundishapur University of Medical Sciences, Ahvaz, Iran; 4 Student Research Committee, Ahvaz Jundishapur University of Medical Sciences, Ahvaz, Iran; 5 Department of Microbiology, Virology and Microbial Toxins, School of Medicine, Guilan University of Medical Sciences, Rasht, Iran; 6 Otorhinolaryngology Research Center, School of Medicine, Guilan University of Medical Sciences, Rasht, Iran; 7 Department of Microbiology, Iran University of Medical Sciences, Tehran, Iran; 8 Department of Microbiology, Faculty of Basic Sciences, Shahrekord Branch, Islamic Azad University, Shahrekord, Iran

**Keywords:** dysbiosis, metabolic health, microbiome, Ramadan fasting, short-chain fatty acids

## Abstract

Ramadan fasting (RF), a culturally embedded form of time-restricted eating, involves daily abstinence from food and drink from dawn to sunset and provides a real-world human model to examine the potential impact of intermittent fasting on the gut microbiome and metabolic health. This review synthesizes evidence from human studies, predominantly observational in design, to evaluate the associations between RF, microbial composition and diversity, and metabolic outcomes. Current evidence suggests that RF may be associated with changes in gut microbial richness and community structure, including reported increases in taxa such *as Akkermansia muciniphila*, *Faecalibacterium prausnitzii, Roseburia*, and *Bacteroides*, many of which are linked to short-chain fatty acid (SCFA) production and intestinal barrier function. However, findings across studies are not entirely consistent, particularly with respect to microbial taxa abundance and SCFA levels. Observational studies also report concurrent changes in anthropometric and metabolic parameters, including body weight, lipid profiles, glycemic markers, and inflammatory indices, although these associations may be influenced by confounding factors such as dietary composition, lifestyle changes, and weight loss during Ramadan. Proposed mechanisms include alterations in feeding–fasting rhythms and microbiota-related pathways such as bile acid metabolism and gut barrier function; however, these mechanisms are largely inferred from related experimental models and should be considered hypothesis-generating in the context of RF. Therefore, while RF represents a relevant ecological model for studying time-restricted eating in humans, the current evidence remains limited by small sample sizes, interindividual variability, and methodological heterogeneity. Further well-controlled, longitudinal, and multi-omics studies are required to clarify causal relationships and determine the extent to which RF-associated microbial changes contribute to metabolic health.

## 1 Introduction

Ramadan fasting (RF) is a widely practiced religious regimen in which individuals abstain from food and drink from sunrise to sunset and resume intake during nighttime hours (1). This month-long practice constitutes a culturally embedded form of time-restricted eating (TRE), where daily caloric intake is confined to a limited feeding window without imposed dietary restrictions (2). Unlike experimental TRE protocols designed for metabolic or weight-management objectives, RF represents a culturally patterned, community-based fasting–feeding cycle characterized by consistent changes in meal timing, frequency, and composition. During Ramadan, dietary patterns often shift toward increased intake of sweets, fruits, and vegetables, alongside reduced consumption of fats, dairy, eggs, and cereals (3).

These unique dietary and behavioral modifications make RF a valuable human model for examining the physiological and microbial consequences of TRE under real-world conditions. The gut microbiota plays a critical role in host metabolism, immune function, and intestinal homeostasis (4). Commensal microorganisms facilitate nutrient metabolism, synthesize essential vitamins and amino acids, and produce short-chain fatty acids (SCFAs) including; acetate, propionate, and butyrate hat support epithelial energy needs and maintain mucosal integrity (5). Disruption of this microbial equilibrium, known as dysbiosis, has been implicated in obesity, insulin resistance, chronic inflammation, and impaired immune responses. The composition and functionality of the gut microbiome are influenced by both diet and feeding fasting rhythms, with circadian patterns shaping microbial diversity and metabolic outputs (2, 6).

In addition to this temporal restriction, RF involves lifestyle and behavioral changes, including altered sleep patterns, meal composition, and daily routines. Therefore, physiological and microbial changes observed before and after Ramadan cannot be attributed solely to fasting itself. These changes likely reflect a combination of fasting and associated lifestyle modifications (7, 8).

Emerging evidence suggests that RF may beneficially modulate gut microbial diversity and community structure. In a pilot study of nine adults, Ozkul et al. (9) reported significantly increased microbial richness after 29 days of RF (*p* = 0.016), along with distinct restructuring of microbial communities based on unweighted UniFrac analysis (*p* = 0.025). Several health-associated genera were enriched following fasting. These taxa are notable SCFA producers and have been linked to improved metabolic and immunological profiles. Detailed microbial signatures observed during RF further support its role as a modulator of gut homeostasis. Studies have reported enrichment of *Akkermansia muciniphila*—an inverse correlate of body weight and a mucin-degrading bacterium resilient to nutrient fluctuations (5, 10, 11).

**Figure 1:**
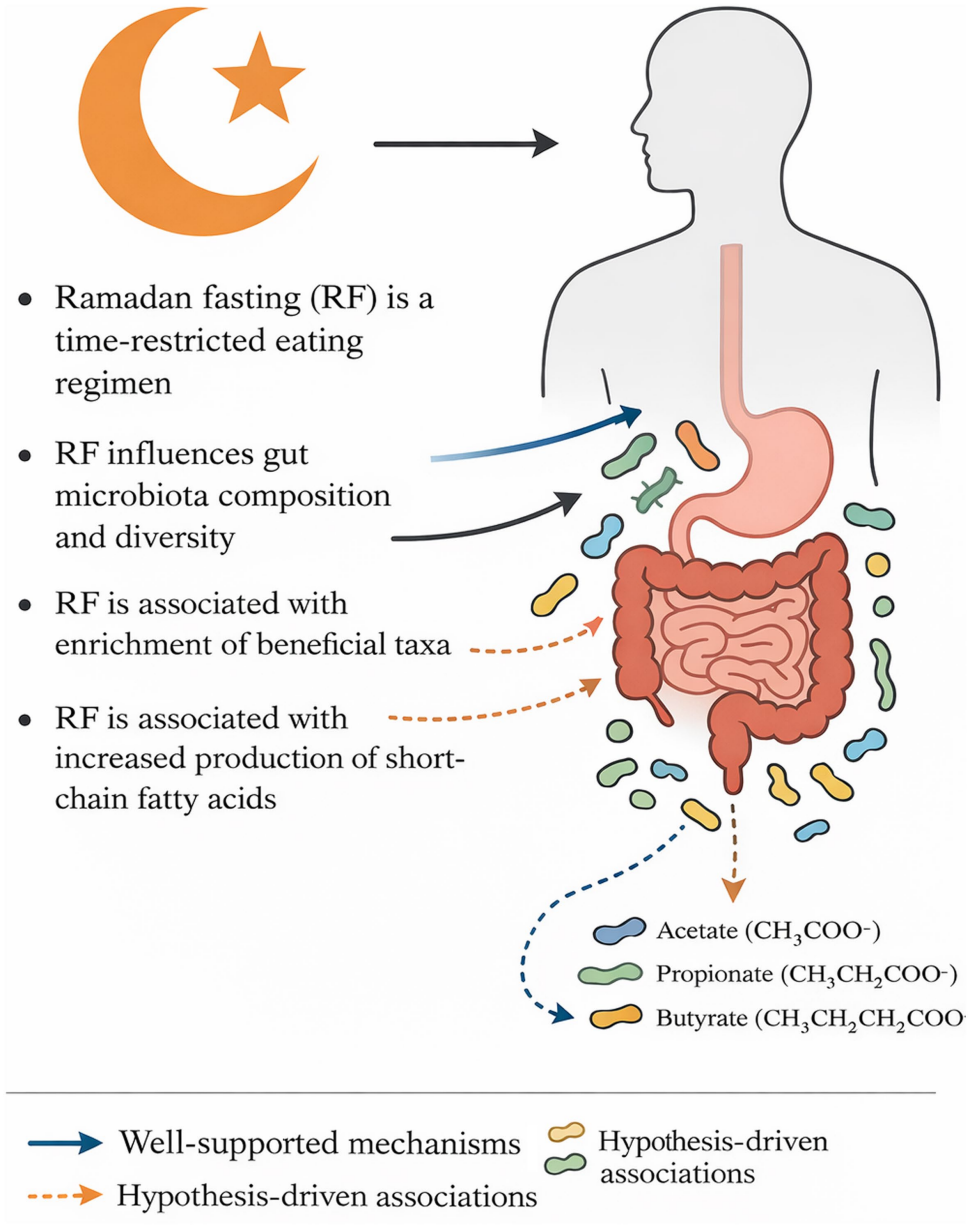
This schematic illustrates RF as a form of diurnal time-restricted eating characterized by daily abstinence from food and drink from dawn to sunset, followed by nocturnal feeding. RF alters feeding rhythms and nutrient availability, which may modulate gut microbial composition and metabolic activity. RF has been associated with changes in gut microbiota diversity and relative abundance of specific taxa, including enrichment of potentially beneficial bacteria. These microbial shifts may contribute to altered production of SCFAs, including acetate (CH_3_COO^−^), propionate (CH_3_CH_2_COO^−^), and butyrate (CH_3_CH_2_CH_2_COO^−^), which play roles in intestinal barrier integrity, host energy metabolism, and inflammatory regulation. Solid arrows represent well-supported mechanisms reported in human studies (e.g., fasting-induced shifts in microbial diversity and SCFA production). Dashed arrows indicate hypothesis-driven or emerging associations inferred from associative human data or supported primarily by experimental and mechanistic studies outside the specific context of RF. Colored microbial icons represent distinct bacterial taxa (schematic only; not taxon-specific). The human silhouette and gastrointestinal tract are symbolic representations. This figure is original and created for the present review.

Other reports describe increased abundance of *Faecalibacterium prausnitzii*, an anti-inflammatory butyrate producer (12), as well as elevated levels of *Butyricicoccus* spp., including *B. pullicaecorum*, known for strengthening intestinal barrier integrity (13) and displaying vulnerability in antibiotic-perturbed microbiota (14). Consistent increases in *Roseburia* considered a hallmark of a healthy gut ecosystem have also been documented, highlighting its role in SCFA production and immune modulation (15). Enrichment of Bacteroides, a metabolically versatile genus capable of switching to host-derived glycans during fasting, further underscores the adaptive microbial responses associated with RF (9). Collectively, these findings suggest that RF may be associated with increased microbial diversity, enrichment of potentially beneficial taxa, and features linked to metabolic resilience (Figure 1).

Given the well-established links between dietary timing, microbial ecology, and host metabolic health, RF may serve as an ecological and culturally embedded strategy for preventing dysbiosis. The objective of this narrative review, supported by a structured search strategy, is to synthesize evidence from human studies to evaluate whether RF, as a model of TRE, is associated with changes in gut microbiota composition and diversity, and whether these microbial alterations may contribute to the prevention of dysbiosis and improvements in host metabolic outcomes, without aiming to meet the methodological criteria of a formal systematic review.

## 2 Methodology

This study was conducted as a narrative review supported by a structured literature search designed to enhance transparency and comprehensiveness, without adhering to formal systematic review or meta-analysis guidelines. This study is a narrative review supported by a structured literature search to ensure transparency and comprehensiveness, without following formal systematic review guidelines. Electronic databases (PubMed/MEDLINE, Scopus, Web of Science, and Google Scholar) were searched for articles published up to 2026. The search strategy combined MeSH terms and free-text keywords related to Ramadan fasting and the gut microbiome, including “Ramadan fasting,” “time-restricted eating,” “intermittent fasting,” “gut microbiota,” “dysbiosis,” and “short-chain fatty acids.” Boolean operators (AND/OR) were used to refine results. Eligible studies included human research on Ramadan fasting or related fasting models that assessed gut microbiota composition, diversity, or metabolic outcomes. Original peer-reviewed English-language articles were included. Animal and *in vitro* studies were excluded unless used for mechanistic context, as were reviews, editorials, and abstracts without primary data. Study selection involved screening of titles and abstracts followed by full-text evaluation of relevant studies.

## 3 Fasting: historical background, definitions, and religious contexts

### 3.1 Brief history of fasting

Fasting is one of the oldest documented human practices, present across diverse cultures prior to organized religions. Early civilizations used fasting for spiritual purification, mental clarity, and ritual preparation. In ancient Greece, philosophers such as Pythagoras and Epimenides associated fasting with mental and spiritual enhancement. Similar practices are described in biblical traditions, including figures such as Moses and John the Baptist (16).

**Figure 2:**
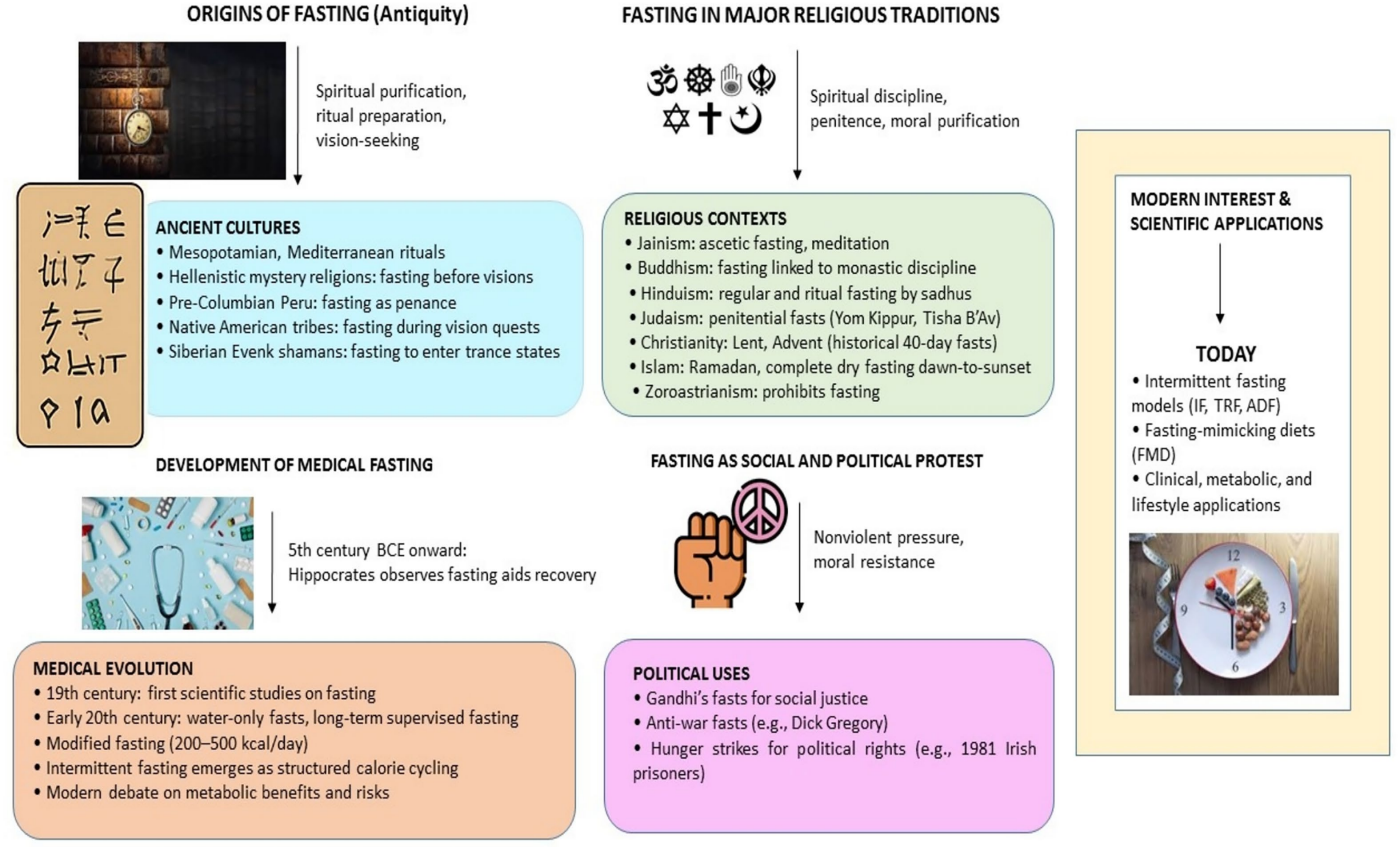
Overview of fasting throughout history, religious traditions, medical evolution, social and political uses, and modern scientific applications (figure prepared using Microsoft PowerPoint).

With the development of major world religions, fasting became formalized and integrated into spiritual doctrine (Figure 2). In Islam, Ramadan represents a month-long period of complete abstinence from food and drink from dawn until sunset, intended to cultivate self-discipline, moral reflection, and spiritual purification (17). Jewish and Christian traditions also incorporate various fasting practices, often linked to penitence, commemoration of historical events, or preparation for major religious observances. Within early Christian monasticism, fasting evolved into a structured ascetic discipline aimed at fostering humility and deepening communion with God (18, 19).

Beyond its religious significance, fasting has a long history as a therapeutic intervention. Ancient medical traditions, including Hippocratic medicine, recommended fasting for the management of acute and chronic illnesses, grounded in the belief that temporary abstinence from food could facilitate bodily restoration (20). This historical perspective has gained renewed relevance in the modern era (Figure 1). Contemporary lifestyles are characterized by prolonged eating windows, caloric excess, and physical inactivity, all of which contribute to rising rates of obesity, type 2 diabetes, and cardiovascular disease, stroke, and neurodegenerative disorders. These epidemiological trends have stimulated growing scientific interest in fasting as a structured dietary strategy aimed at improving metabolic health and reducing disease risk (18, 20).

Scientific studies in the 19th and 20th centuries explored metabolic responses to fasting, including ketosis and energy adaptation. Physicians, writers, and early metabolic researchers documented prolonged fasts, using them as opportunities to examine physiological responses to starvation, including shifts in energy metabolism and adaptations in glucose and fat utilization. These early observations laid the groundwork for modern studies on fasting metabolism, ketogenesis, and the therapeutic potential of dietary restriction (16).

Fasting has also functioned as a powerful social and political tool. Hunger strikes led by figures such as Mahatma Gandhi and Irish political prisoners illustrate its historical role in nonviolent resistance and moral protest (16, 21). Altogether, fasting represents a deeply rooted human practice shaped by interwoven religious, medical, cultural, and political traditions. Its contemporary scientific relevance continues to expand, particularly as researchers explore its potential benefits for metabolic regulation, disease prevention, and overall health.

### 3.2 Definition and religious contexts of fasting

Fasting is the voluntary restriction of food, drink, or both for a defined period and is practiced for spiritual, cultural, and therapeutic purposes (22). It represents a deliberate departure from ordinary eating patterns and induces a characteristic metabolic shift involving glycogen depletion, increased lipolysis, ketone-body production, and activation of cellular stress-response pathways (23). Historically, fasting has played a central role in many religious traditions: in Islam, Ramadan involves complete abstinence from food and drink from dawn to sunset for 29–30 days; in Judaism, major fasts such as Yom Kippur and Tisha B’Av require approximately 25 h of abstinence; in Christianity, practices such as Lent include partial fasting or avoidance of particular foods as acts of penance; in Buddhism, monastic communities typically refrain from solid food between noon and dawn to cultivate mindfulness; and in Hinduism, observances such as Ekadashi and Navratri involve avoiding grains or specific foods as a form of purification and devotion (18, 19). Beyond its religious significance, fasting has long been used in medical traditions, including Hippocratic medicine, as a therapeutic strategy, and is now studied extensively as a structured dietary intervention in response to modern patterns of caloric excess and the rising prevalence of metabolic disease (20).

### 3.3 Classification and mechanisms of fasting

#### 3.3.1 Time-restricted feeding (TRF)

TRF involves limiting nutrient intake to a defined daily window (typically 4–12 h) without overall caloric restriction (Table 1). Religious practices such as RF resemble experimental TRF in animals and TRE in humans (these terms are sometimes used interchangeably in the literature), as both share the principle of temporal restriction of food intake. However, unlike controlled TRF or TRE protocols, RF encompasses additional lifestyle and behavioral factors, making its physiological effects more complex and context-dependent (24). TRF has been shown to synchronize feeding–fasting cycles with circadian rhythms under controlled experimental conditions, thereby influencing metabolic and endocrine functions (24, 25). Molecularly, TRF entrains peripheral clocks in organs such as the liver, adipose tissue, and muscle, modulating the expression of core circadian genes (Bmal1, Clock, Per, Cry) and metabolic regulators including AMPK, AgRP, and CREB (24). TRF has demonstrated beneficial effects on insulin sensitivity, lipid metabolism, energy homeostasis, and gut microbiota rhythmicity (24–26). Moreover, it influences appetite regulation via hypothalamic neurons (NPY/AgRP and POMC/CART) and hormones such as leptin, ghrelin, and insulin (24, 27). Overall, TRF’s health benefits are mediated largely through circadian alignment and modulation of temporal gene expression.

**Table 1:** Major types of fasting and their characteristics.

Main category	Type of fasting	Description	Duration	Common context	References
Time-Restricted/intermittent fasting	Intermittent fasting (IF)	Alternating periods of fasting and eating; induces glucose-to-ketone switch, affects metabolism, immunity, and gut microbiota	Hours per day	General health, research	(68)
	Alternate-Day Fasting (ADF)	Fasting every other day; partial (≈25% caloric intake) or complete; improves metabolic parameters	24 h	Weight management	(95)
	Ramadan Intermittent Fasting (RIF)	Abstinence from food and drink from dawn to sunset; an ecological time-restricted eating model with a consistent daily feeding schedule	29–30 days	Religious, cultural	(68, 96)
	Time-Restricted Eating (TRE)	Daily fasting window (e.g., 16/8, 18/6); feeding restricted to specific hours without overall caloric restriction	4–12 h per day	Health, research	(68)
Prolonged / Periodic Fasting	Periodic/Prolonged Fasting	Continuous fasting for multiple days; induces ketogenesis, autophagy, and metabolic adaptation	2–28 days	Therapeutic, supervised	(97)
	Water-Only Fasting	Complete abstinence from all nutrients except water; used in clinical or therapeutic contexts	1–28 days	Therapeutic, clinical	(97)
Fasting-Mimicking Diet (FMD)	Fasting-Mimicking Diet	Low-calorie, plant-based diet mimicking prolonged fasting; maintains nutrient supply; promotes immune, endocrine, and stem cell benefits	5 consecutive days/month	Health, rejuvenation, research	(18)

#### 3.3.2 Intermittent fasting

IF encompasses alternating periods of normal eating and fasting, ranging from hours to entire days. Common protocols include alternate-day fasting (ADF), the 5:2 diet, and TRE (Table 1). IF induces a metabolic switch from glucose to ketone utilization (glucose-to-ketone switch), reduces circulating glucose, insulin, and IGF-1, and promotes lipolysis and ketogenesis (24, 28, 29). Beyond metabolism, IF modulates gut microbiota composition, increasing beneficial microbial families (e.g., *Lactobacillaceae, Bacteroidaceae, Firmicutes*) and short-chain fatty acid production, thereby influencing systemic immunity and interorgan communication (30, 31). IF also affects peripheral organs, including the liver, pancreas, adipose tissue, and stem cells, and is emerging as a regulator of noncoding RNAs involved in metabolic and mitochondrial pathways (24).

#### 3.3.3 Fasting-mimicking diet

FMD is a low-calorie, plant-based dietary protocol that mimics prolonged fasting while maintaining nutrient supply (Table 1). Typically, caloric intake is restricted for five consecutive days per month (≈1,090 kcal on day 1, 725 kcal on days 2–5). FMD promotes immune system rejuvenation, enhancing lymphocyte circulation, antitumor immunity, and hematopoietic stem cell regeneration (24, 32, 33). FMD also benefits the endocrine and nervous systems, promoting *β*-cell regeneration, neuronal differentiation, oligodendrocyte progenitor cell maturation, and intestinal stem cell function (24). Mechanistically, FMD activates AMPK, suppresses mTOR signaling, and promotes autophagy and cellular repair processes, thus replicating the physiological benefits of complete fasting while improving adherence (24, 34).

Collectively, fasting is a deeply rooted human practice shaped by religious, cultural, medical, and political traditions. Modern scientific inquiry has reframed fasting not only as a spiritual discipline but also as a structured metabolic intervention capable of influencing circadian biology, hormonal signaling, immune modulation, and cellular stress-response pathways. Among contemporary fasting paradigms, RF occupies a unique position as a culturally embedded, time-restricted eating model that integrates behavioral and physiological adaptations. Understanding these historical and mechanistic foundations provides essential context for interpreting its potential microbiome-mediated metabolic effects.

## 4 Microbiome and dysbiosis

### 4.1 The healthy gut microbiome

The gut microbiome is a complex and dynamic community of microorganisms including bacteria, fungi, viruses, archaea, protozoa, and their metabolites, all living in close interaction with the host. This ecosystem profoundly influences human health (35). Among bacteria, four phyla dominate the intestinal environment: *Firmicutes*, *Bacteroidetes*, *Actinobacteria*, and *Proteobacteria* (35, 36). While each individual has a unique microbiome, early-life factors such as mode of birth, breastfeeding, and host genetics shape its foundational composition (37). Across the lifespan, diet, age, environment, stress, medications, and lifestyle continuously fine-tune the microbial community, affecting metabolism, immunity, and overall health (35, 38, 39).

A balanced gut microbiome plays a crucial role in maintaining metabolic and immune homeostasis. It supports nutrient digestion, ferments otherwise indigestible fibers, and produces essential metabolites such as SCFAs and vitamins (40). These microbial products also regulate immune function by activating macrophages, promoting IgA secretion, and modulating systemic immunoglobulin synthesis (39). Unsurprisingly, disruption of this delicate microbial ecosystem has been linked to a range of diseases, particularly metabolic disorders including obesity, type 2 diabetes, metabolic syndrome, cardiovascular disease, and metabolic dysfunction–associated steatotic liver disease (MASLD) (41, 42). Interventions, dietary, surgical, pharmacologic, or lifestyle-based can significantly shift microbiota composition, often altering the *Firmicutes*/*Bacteroidetes* balance and hundreds of other microbial taxa, further underscoring the intricate link between the microbiome and metabolic health (43).

### 4.2 Dysbiosis and metabolic health

Dysbiosis refers to a pathological imbalance in the gut microbiome, often resulting from poor diet, lifestyle factors, medications, or genetic predispositions. This imbalance disrupts normal host–microbiota interactions and contributes to a variety of metabolic disturbances (44).

#### 4.2.1 Gut barrier dysfunction

A key consequence of dysbiosis is compromised gut barrier integrity. A weakened barrier allows microbial components such as lipopolysaccharides (LPS) to enter circulation, triggering systemic inflammation (45). Markers of barrier dysfunction include elevated serum zonulin, LPS, and endotoxin levels (35). Certain microbes help maintain barrier integrity: *A. muciniphila* promotes mucus turnover and epithelial regeneration, while its protein Amuc_1100 reduces cellular stress and prevents leakage (46, 47). *Lactobacillus* species enhance expression of MUC2 and tight junction proteins (ZO-1, JAM-1) (48, 49). *Bifidobacterium* species strengthen occludin and ZO-1 (50). *Faecalibacterium prausnitzii* supports junction regulation via metabolic signaling (51).

#### 4.2.2 Metabolic endotoxemia and inflammation

Barrier dysfunction can lead to metabolic endotoxemia, where circulating microbial components activate Toll-like receptors (TLR2 and TLR4) on immune, endothelial, adipocyte, and hepatic cells. This triggers inflammatory pathways (NF-κB and JNK), promoting production of cytokines such as TNF-*α* and IL-6 (52). Chronic inflammation then interferes with insulin signaling, impairs *β*-cell function, and contributes to insulin resistance and metabolic disease (35, 53). Transplant studies confirm that dysbiotic microbiota can transfer these metabolic disturbances to healthy hosts (54, 55).

#### 4.2.3 Bile acid dysmetabolism

Dysbiosis alters bile acid (BA) metabolism and impairs FXR/TGR5 signaling that regulate glucose homeostasis, lipid metabolism, and cholesterol processing. BAs are synthesized in the liver from cholesterol and conjugated with glycine or taurine before being secreted into the intestine, where they aid in lipid absorption and are extensively recycled via the enterohepatic circulation (56). The gut microbiota plays a central role in BA transformation, primarily through the activity of bile salt hydrolase (BSH), which deconjugates BAs, and other microbial enzymes that perform dehydroxylation, dehydrogenation, and desulfation, generating secondary and tertiary BA metabolites (56, 57). These microbial BA transformations are critical for the activation of key BA receptors such as farnesoid X receptor (FXR), G protein-coupled BA receptor (TGR5), vitamin D receptor (VDR), and pregnane X receptor (PXR), which regulate glucose, lipid, and energy metabolism, as well as immune responses (56). Dysbiosis, particularly a reduction in BSH-active bacteria, impairs these transformations, leading to altered receptor signaling, decreased BA diversity, and accumulation of conjugated BAs that cannot fully activate FXR or TGR5. As a result, metabolic dysregulation can occur, including impaired glucose tolerance, dyslipidemia, and disrupted cholesterol homeostasis, forming a mechanistic link between microbial imbalance and the progression of metabolic disorders (56). Moreover, BA dysmetabolism contributes to chronic low-grade inflammation, gastrointestinal barrier dysfunction, and altered production of incretins such as GLP-1, GLP-2, and GIP, further impacting insulin sensitivity and energy homeostasis. Therapeutic strategies aimed at restoring BA metabolism, such as BSH-active probiotics or modulation of gut microbiota composition, are being explored to improve metabolic outcomes in obesity, type 2 diabetes, and cardiovascular diseases (56, 58). Importantly, although gut microbiota–bile acid interactions are well established in human metabolic studies, direct evidence linking RF specifically to modulation of bile acid signaling pathways remains limited. Therefore, the relevance of these mechanisms in RF should be interpreted in light of broader diet–microbiome and fasting literature, where bile acids act as key mediators of host–microbiota metabolic crosstalk (59).

### 4.3 Microbial metabolites and metabolic regulation

SCFAs, acetate, propionate, and butyrate are produced by fermentation of dietary fibers and act as key messengers between microbes and the host (35). SCFAs strengthen gut barrier function and modulate inflammatory responses such as TNF-*α* and IL-1*β* (60). Other microbial metabolites, such as succinate, also maintain epithelial integrity and modulate inflammation, highlighting the broad metabolic influence of the gut microbiota (61). Human and animal studies show that higher SCFA levels correlate with improved insulin sensitivity, lower visceral fat and better β-cell function, although some variability exists (62–66). Recent findings highlight RF as a physiologically relevant model of intermittent fasting capable of modulating the gut microbiota. Unlike other forms of fasting such as alternate-day fasting (41), complete alternate-day fasting (67), or TRE (68), RF involves a daily fasting interval (typically 14–16 h from dawn to sunset) followed by a controlled feeding window. This structured pattern has been shown to influence gut microbial composition, promoting the growth of SCFA-producing bacteria and enhancing gut barrier integrity (41). These microbiome-modulating effects are consistent with mechanistic pathways linking dysbiosis, bile acid metabolism, and barrier dysfunction to host metabolic homeostasis (69, 70).

In addition to microbiota-host interactions discussed above, nutrient-sensing signaling pathways such as mechanistic target of rapamycin complex 1 (mTORC1) represent important regulators of systemic metabolic responses to feeding and fasting rhythms. In addition to microbiota-mediated pathways, nutrient-sensing signaling networks such as mTORC1 represent key regulators of metabolic adaptation to feeding–fasting cycles. mTORC1 integrates signals from amino acids, glucose, and growth factors to regulate anabolic metabolism, while its downregulation during fasting promotes autophagy and metabolic flexibility. Evidence from human and experimental intermittent fasting studies indicates that modulation of mTOR signaling contributes to improved metabolic homeostasis and cellular stress resistance. However, it should be noted that direct evidence linking RF specifically to mTOR pathway modulation in humans remains limited. Therefore, the involvement of mTOR-related mechanisms in RF should be considered biologically plausible and hypothesis-generating, providing a mechanistic framework to interpret observed metabolic and microbiome-associated changes (71–74).

Dysbiosis disrupts these pathways, contributing to chronic inflammation, insulin resistance, and cardiometabolic disease progression. RF may influence these mechanisms through microbial shifts that are associated with increased SCFA production, enhance barrier stability, and modulate bile acid receptor signaling. While current evidence is largely associative, the mechanistic framework linking microbial ecology to host metabolic regulation provides a strong biological rationale for examining RF as a modulator of dysbiosis-related pathways. It should be noted that most evidence on RF is derived from observational studies conducted during Ramadan, in which concurrent changes in caloric intake, diet composition, sleep patterns, and physical activity may contribute to the observed metabolic and microbiome-related effects.

## 5 The role of the RF on microbiome, metabolic, and biochemical changes

### 5.1 Studies primarily examining gut microbiota composition and diversity

RF has been reported to be associated with remodeling of gut microbiota composition, with these changes correlating to favorable physiological shifts such as reduced body weight and improved blood parameters (75, 76).

In a study of 30 healthy adults, Su et al. documented alterations in the gut microbiome occurring during RF. Their findings showed that this fasting regimen enhanced microbiome diversity, particularly marked by an increase in bacterial families *Lachnospiraceae* and *Ruminococcaceae*, which belong to the Clostridiales order. After fasting cessation, microbiome composition returned to baseline. Additionally, fluctuations in *Lachnospiraceae* levels closely reflected the physiological changes induced by intermittent fasting. Regarding the fasting cohort in the study, there was a notable decrease in body weight alongside favorable metabolic shifts in blood parameters, whereas the non-fasting participants exhibited relatively stable measurements throughout the study duration. Specifically, body weight in the fasting group significantly dropped from before fasting (T1) to the end of fasting (T2) and remained reduced even 30 days post-fasting (T3). There was also a tendency toward reduced body fat mass. Liver enzymes generally remained stable, with *γ*-glutamyltransferase (GGT) levels significantly lowering during fasting. Blood glucose slightly decreased, although glycated hemoglobin (HbA1C) remained unchanged. Serum lipid profiles were only mildly variable, with triglycerides notably declining between T2 and T3. Markers of kidney function (creatinine, urea, uric acid) showed modest but statistically significant changes during fasting, indicating metabolic adaptations. The intermittent fasting-driven increase in butyric acid-producing *Lachnospiraceae* likely provides a mechanistic basis for the health benefits associated with this fasting regimen (76). It should be noted that the reported associations between microbial shifts and metabolic parameters were based primarily on observational correlations within the study, and adjustment for potential confounders such as body weight change was limited/not consistently reported.

An initial investigation into intermittent fasting found that RF results in a higher presence of *Akkermansia muciniphila* and the *Bacteroides fragilis* group. Following RF, all participants exhibited a significant increase in the abundance of both *A. muciniphila* and the *B. fragilis* group compared to their baseline measurements (*p* = 0.004 and 0.008, respectively). Additionally, serum fasting glucose and total cholesterol concentrations showed a significant decrease across all subjects (*p* < 0.01 and *p* = 0.009, respectively) (77).

Saglam et al. investigated the effects of RF on gut microbiota composition in 12 healthy adults. They assessed the relationships between participants’ dietary intake and the relative abundances of various bacterial genera. Their findings indicated that, prior to fasting, *Firmicutes* were more prevalent in the gut microbiome, but their levels declined significantly by the end of Ramadan (*p* < 0.05). In contrast, *Proteobacteria* showed a marked increase after the fasting period (*p* < 0.05). Fasting was also linked to notable reductions in seven genera—*Blautia*, *Coprococcus*, *Dorea*, *Faecalicatena*, *Fusicatenibacter*, *Lachnoclostridium*, and *Mediterraneibacter*—whereas *Escherichia* and *Shigella* became more abundant following Ramadan. Analysis of dietary intake revealed three significant negative correlations: Ihubacter with protein consumption (rho = −0.54, *p* = 0.0068), *Fusicatenibacter* with vegetable intake (rho = −0.54, *p* = 0.0042), and *Intestinibacter* with nut consumption (rho = −0.54, *p* = 0.0065). Therefore, the study suggests that although the fasting schedule during Ramadan remains uniform, individual dietary choices can still shape the structure of the gut microbiota. Given that dietary composition is a primary determinant of gut microbial structure, these associations further support the role of Ramadan-associated dietary shifts in modulating microbiota composition and subsequent metabolic responses (78). These correlations were based on observational analyses, and the extent to which potential confounders such as total energy intake or body weight were statistically controlled was not clearly detailed.

A separate investigation found that RF reshapes the human gut microbiome in ways that reflect dietary patterns. In this study, assessments of alpha diversity revealed that fasting during Ramadan markedly changed the coverage and ACE indices in Chinese participants, while no comparable alterations were detected in either index for the other cohort. In the Pakistani group, the dominance of *Bacteroidetes* and *Firmicutes* was largely driven by *Prevotella* and *Faecalibacterium*, whereas among Chinese subjects, *Bacteroides* (within Bacteroidetes) remained the leading genus both before and after the fasting period. We identified notable increases in certain taxa and reductions in others across individuals from both populations, indicating that fasting may influence beta diversity. After fasting, *Dorea*, *Klebsiella*, and *Faecalibacterium* became more abundant in the Chinese cohort, whereas *Sutterella*, *Parabacteroides*, and *Alistipes* showed significant enrichment in the Pakistani cohort. When the two groups were analyzed together, the genera *Coprococcus*, *Clostridium*_XlV, and *Lachnospiracea* all showed significant declines following the fasting interval. Dietary assessment demonstrated that energy intake from fats had a positive correlation with *Oscillibacter* and *Prevotella*, but an inverse relationship with Bacteroides. Additionally, higher consumption of sweets was strongly associated with increased levels of *Akkermansia*. These findings suggest that diet is the primary determinant of gut microbiota composition and aligns closely with ethnic background, while fasting results in the enrichment of certain bacterial groups in specific individuals (79).

A pilot investigation involving 20 healthy adults explored how RF influences the gut microbiome, offering new insights into its physiological effects. The study found reductions in short-chain fatty acids—specifically acetate, butyrate, and propionate—along with declines in beneficial bacterial populations during Ramadan. At the same time, microbial diversity increased after Ramadan, implying that the dietary patterns followed during the fasting month may not fully sustain a healthy gut microbial environment. Among Muslim participants observing the fast, the relative abundances of major phyla such as *Bacteroidota* and *Firmicutes* differed from person to person, as did the proportions of families including *Lachnospiraceae*, *Prevotellaceae*, *Ruminococcaceae*, and *Bacteroidaceae*. Notably, small amounts of *Spirochaetota*, a group that contains some pathogenic species, were also identified in the microbial profiles of certain individuals. Throughout the study period—both during fasting and afterward—most participants consistently exhibited bacterial communities dominated by genera such as *Prevotella*, *Blautia*, and *Faecalibacterium*. Moreover, pronounced differences in functional gene profiles based on metagenomic data, together with the strong association observed between *Lactobacillus* and SCFA levels, further reinforce the study’s main hypothesis (80). Based on the observed operational taxonomic unit (OUT) measurements, microbial richness increased significantly after Ramadan (*p* = 0.016). However, no meaningful changes were detected in alpha diversity when evaluated using the Shannon index or phylogenetic diversity measures. LEfSe analysis showed that several genera—including *Butyricicoccus*, *Bacteroides*, *Faecalibacterium*, *Roseburia*, *Allobaculum*, *Eubacterium*, *Dialister*, and members of *Erysipelotrichi*—were markedly enriched once the fasting period concluded. Random forest modeling further indicated that *Butyricicoccus pullicaecorum* was the species most strongly influenced by RF. Collectively, these findings demonstrate that RF, a form of intermittent fasting, induces notable shifts in gut microbiota composition (9).

A study examining the impact of RF on intestinal microbiota and fatty acid binding protein 4 (FABP4) in 10 individuals with overweight and obesity found that both alpha and beta diversity increased significantly after the fasting period (*p* < 0.05). Notable reductions were observed in the *Firmicutes*/*Bacteroidetes* ratio, the *Firmicutes* phylum, the Clostridia class, the Clostridiales order, and the *Ruminococcaceae* family. In contrast, the Bacteroidetes and Proteobacteria phyla, along with the Bacteroidia, Alphaproteobacteria, and Erysipelotrichi classes, and the Bacteroidales, Erysipelotrichales, and Actinomycetales orders, as well as the *Erysipelotrichaceae* family and the genus *Prevotella*, showed significant elevations (*p* < 0.05). Participants who lost an average of 2.3 ± 0.99 kg during Ramadan experienced an increase in HDL-C and a reduction in triglyceride levels (*p* < 0.05). Although FABP4 concentrations declined post-fasting, the change did not reach statistical significance (*p* > 0.05). Overall, RF promotes weight reduction, alters gut microbial composition, and enhances lipid parameters and FABP4 trends (81).

In 2025, researchers examined gut microbiome remodeling in six Pakistani expatriates residing in China who observed RF. The study revealed a notable increase in alpha diversity throughout the RF period, with gut microbial structural changes becoming more pronounced by the end of RF compared to the beginning. Significant inter-individual variability was observed, with marked alterations in several bacterial species such as *Clostridium perfringens*, *Coprococcus comes*, and *Lactococcus lactis* (*p* < 0.05). After the conclusion of RF, noticeable shifts were detected in the relative abundance of specific bacterial taxa, including *Actinomyces* sp. ICM47, *Bifidobacterium catenulatum*, *Faecalibacterium prausnitzii*, *Firmicutes bacterium* CAG95, *Ruminococcus callidus*, *Slackia isoflavoniconvertens*, and various *Streptococcus* species, with changes mostly appearing at the second or third post-fasting time points (82). Variability is observed in the relative abundance of specific microbial taxa across studies. Such differences are likely driven by heterogeneity in study populations, baseline microbiome composition, geographic and cultural dietary patterns, and the analytical approaches used for microbial profiling.

### 5.2 Studies primarily examining metabolomic, immunologic, and biochemical pathway changes

Beyond compositional changes in the gut microbiota, RF has been associated with alterations in circulating metabolic markers. Although these findings are derived from blood-based analyses, they are highly relevant to gut health due to the well-established bidirectional interactions between the gut microbiota and host metabolic regulation, as demonstrated in a study of 25 healthy adults. Microbial-derived metabolites such as SCFAs, secondary bile acids, and other bioactive compounds influence lipid metabolism, glucose homeostasis, adipokine signaling, and inflammatory pathways through gut–liver and gut–adipose axis mechanisms. Therefore, systemic metabolic changes observed during RF may represent downstream physiological consequences of microbiota modulation. In the prospective cohort study that evaluated metabolomics changes during Ramadan diurnal intermittent fasting (RDIF), metabolomic profiling was performed on plasma samples collected from participants before and at the end of the fasting month. This analysis involved 25 metabolically healthy adults with overweight and obesity (7 females, 18 males, mean age 39.48 ± 10.0 years) and employed untargeted liquid chromatography–mass spectrometry (LC–MS) to identify differences in metabolite levels between the two time points. The evaluation showed that 27 metabolites exhibited significant differences (*p* < 0.05) between pre- and post- RDIF periods. Of these, 23 metabolites declined during fasting, encompassing amino acids like aspartame, tryptophan, phenylalanine, and histidine, as well as others such as valeric acid and cortisol. Conversely, just four metabolites rose post-RDIF, namely traumatic acid, 2-pyrrolidinone, PC [18:1(9Z)/18:1(9Z)], and L-sorbose. Compared to baseline, the end of RF was associated with marked reductions in body weight, waist circumference, and LDL cholesterol, alongside rises in fasting blood glucose (remaining within normal limits) and HDL cholesterol. Analysis via the MetaboAnalyst® platform highlighted key enriched pathways: (1) histidine metabolism; (2) folate biosynthesis; (3) phenylalanine, tyrosine, and tryptophan biosynthesis; (4) aminoacyl-tRNA biosynthesis; (5) caffeine metabolism; (6) vitamin B6 metabolism; plus, various lipid-related pathways including arachidonic acid metabolism, glycerophospholipid metabolism, and linoleic acid metabolism. Therefore, RDIF induces substantial shifts across multiple metabolic pathways, mirroring the distinct dietary and lifestyle modifications during the fasting period (83).

The 2025 study of six healthy Pakistani expatriates also reported that RF significantly influenced metabolic pathways related to amino acids, carbohydrates, energy production, glycan biosynthesis, and the metabolism of cofactors and vitamins. Key metabolites such as pyridoxamine, glutamate, citrulline, arachidonic acid, and short-chain fatty acids exhibited substantial variation across different time points according to predicted metabolic pathways (82).

An investigation into the impact of RF on chronic inflammatory markers and gut-derived endotoxins in Egyptian hemodialysis patients included 27 individuals who fasted for more than 15 days (average 29 ± 2.2 days). Following the fasting period, significant reductions were recorded in high-sensitivity C-reactive protein (hsCRP) (median 62 mg/L vs. 91 mg/L), trimethylamine-N-oxide (TMAO) (median 4.5 μmol/L vs. 17 μmol/L), the platelet-to-lymphocyte ratio (PLR) (mean 98.9 vs. 111.8), and the neutrophil-to-lymphocyte ratio (NLR) (median 1.56 vs. 1.59), with *p*-values < 0.001, < 0.001, < 0.001, and 0.04, respectively. These findings indicate that RF exerts a favorable influence on bacterial endotoxin levels as well as markers of chronic inflammation in patients undergoing hemodialysis (84).

Hassanein et al. conducted a study examining biometric and metabolic changes in diabetic patients before, during, and after Ramadan. The study recruited 342 adult participants with low-to-moderate risk type 2 diabetes (T2DM), classified as having mild to moderate medical risk according to established clinical fasting risk stratification guidelines. Importantly, participants had independently chosen to fast during Ramadan, and the study was observational in nature, conducted with institutional ethical approval and appropriate medical oversight. The cohort was comprised of 52.3% males (*n* = 180) and 47.7% females (*n* = 162). Most outcomes exhibited a U-shaped pattern across the pre-Ramadan, Ramadan, and post-Ramadan periods, with a slight but statistically significant weight reduction during fasting that returned to baseline after Ramadan. The findings indicate that fasting does not significantly increase risks related to glycemic control, body weight, or blood pressure in many diabetic individuals. Therefore, the results support current recommendations permitting low-risk diabetic patients to fast during Ramadan or other days as they wish (85).

A study investigated the impact of 30 days of RF on the autophagy pathway and metabolic health in 50 healthy individuals. The controlled cohort included 50 participants aged 20 to 78 years, with 24 fasting and 26 non-fasting subjects. Blood samples collected at the end of Ramadan were analyzed for biochemical, hematological, and inflammatory markers, including serum IL-6 and hs-CRP levels. Autophagy markers were assessed through serum proteins (Beclin-1 and LC3β) using ELISA and gene expression of Beclin-1, p62, and LC3β via real-time PCR. No significant changes were observed in biochemical parameters (except BUN), inflammatory markers, or hematological indices during Ramadan. Notably, Beclin-1 gene expression increased significantly in the fasting group, indicating autophagy initiation, while LC3β and p62 levels decreased in peripheral blood mononuclear cells. Additionally, fasting women showed significantly elevated serum Beclin-1 levels. Overall, RF did not adversely affect biochemical, hematological, or inflammatory markers and may activate autophagy pathways to adapt to energy and nutrient restriction during fasting (86). Collectively, while many of these outcomes are assessed through circulating biomarkers, they should not be interpreted as isolated systemic effects. Rather, they may reflect integrated host–microbiome metabolic adaptations occurring during Ramadan fasting, mediated through gut-derived metabolites, altered substrate availability, and gut–organ axis communication. Notably, findings across studies are not entirely consistent with respect to SCFA levels, with some reports indicating increases while others describe reductions or no significant change following Ramadan fasting. These discrepancies may reflect differences in dietary fiber intake, timing of sample collection, host metabolic status, and methodological variability in metabolite quantification.

### 5.3 Studies primarily examining anthropometric and cardiometabolic changes in healthy individuals

The impact of RF on clinical and biochemical markers was examined in 92 healthy adults. During fasting, men experienced a notable decrease in body weight and body mass index (BMI), while women showed a significant reduction in waist circumference. Blood pressure, including both systolic and diastolic measures, remained unchanged throughout the fasting period. Fasting plasma glucose levels significantly dropped in both men and women (*p* < 0.0001), with no cases of hypoglycemia reported among participants. In females, a significant positive correlation was observed between fasting serum glucose and total caloric intake (*p* = 0.001), indicating that higher caloric intake was associated with elevated fasting glucose levels. Improvements in lipid profiles were observed for both genders, with decreases in serum total cholesterol, triglycerides, and LDL cholesterol, along with a significant rise in HDL cholesterol. In women, calorie reduction correlated positively with increased HDL cholesterol (*p* < 0.03), and in men, lower calorie intake showed a trend toward reduced LDL cholesterol (*p* = 0.08). Both men and women significantly reduced their total daily caloric consumption during Ramadan. The mean duration of the daily fasting period was 11.5 ± 0.5 h (87).

A study of 30 healthy adults by Saleh et al. revealed that weight and BMI remained relatively stable throughout Ramadan, with no significant changes observed. Nevertheless, there were notable decreases in insulin and leptin levels, accompanied by a significant increase in ghrelin (*p* ≤ 0.05). Fasting also triggered significant alterations in antioxidant enzyme activity and lipid peroxidation (LPO) (*p* ≤ 0.05). All measured lipid parameters were influenced by fasting; triglycerides, total cholesterol, LDL-C, VLDL-C, CH/HDL-C ratio, TG/HDL-C ratio, LDL-C/HDL-C ratio, TyG index, and HOMA-IR demonstrated significant declines, whereas HDL-C levels increased significantly (*p* ≤ 0.05). Lifestyle and dietary changes during RF likely contributed to these effects (88).

A prospective cohort study of 62 Saudi women assessed the influence of RF on anthropometric, hormonal, metabolic, inflammatory, and oxidative stress indicators in both pre- and post-menopausal participants. At Ramadan’s conclusion, both groups showed significant reductions (*p* < 0.05) compared to pre-fasting in body weight, BMI, waist circumference, body fat percentage (BFP), fat mass, fat mass index, triglycerides, and diastolic blood pressure. In contrast, HDL, SOD, GPx, and IL-10 levels rose significantly (*p* < 0.05) in both cohorts. Estrogen concentrations dropped markedly (*p* < 0.05) in pre-menopausal women but increased significantly (*p* < 0.05) in post-menopausal women, while progesterone, TAC, MDA, and IGF-1 remained stable across groups. TNF-*α* declined significantly in both, though more substantially in pre-menopausal women. Sex hormones and select metabolic markers displayed varied positive or negative correlations with BMI and BFP, particularly in post-menopausal women. RF likely modulates estrogen, TNF-α, and IL-10 via enhanced metabolic health, fat loss, autophagy activation, immune modulation, and hormonal shifts. Hence, it correlated with beneficial changes in anthropometric, metabolic, inflammatory, and oxidative stress profiles for healthy pre- and post-menopausal women (89).

Analyses from another study involving 15 athletes indicated that RF resulted in significantly lower post-fasting levels of weight, body fat mass, neutrophils, hematological inflammatory markers, cholesterol, triglycerides, and low-density lipoprotein. Trends toward decreases were observed in body mass index, body fat percentage, and lean body mass, and blood volume, whereas lymphocytes increased significantly. Erythrocyte indices and kidney function tests remained unchanged. These results suggest that RF promotes weight reduction, enhances cardiovascular health, and alters immune response patterns in athletes. Additional research is needed to clarify the underlying mechanisms (90).

**Figure 3:**
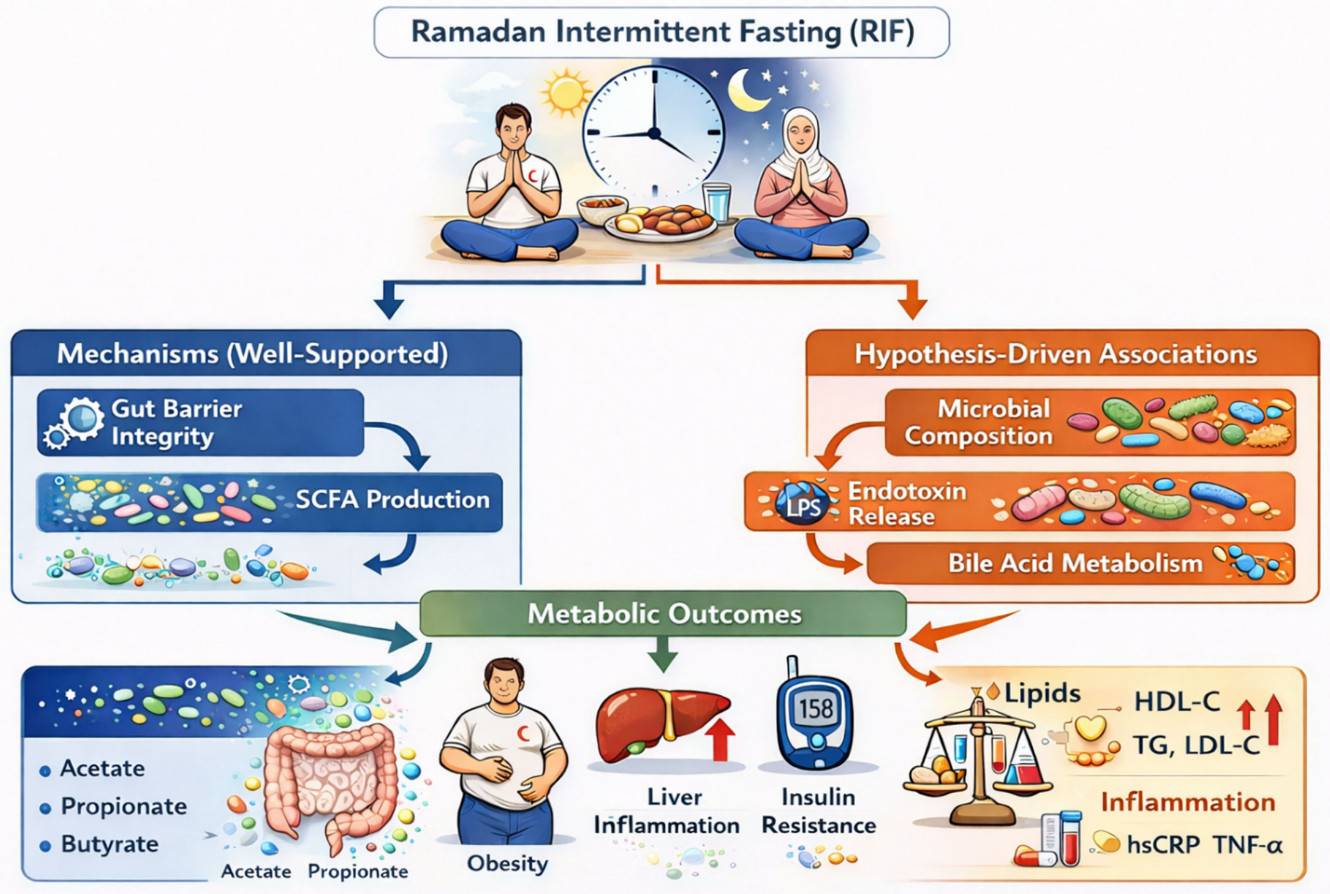
Proposed mechanistic framework linking RF–induced feeding rhythms to gut microbiota alterations and downstream metabolic outcomes. RF represents a diurnal time-restricted feeding pattern that alters nutrient timing and availability. Well-supported mechanisms (solid arrows, blue panel) include improved gut barrier integrity and altered SCFA production (acetate, propionate, and butyrate). Hypothesis-driven or emerging associations (dashed arrows, orange panel) include changes in microbial composition, LPS release, and bile acid metabolism. These pathways may contribute to systemic metabolic outcomes (green panel), including alterations in lipid profile (HDL-C, TG, LDL-C), inflammatory markers (hsCRP, TNF-α), insulin resistance, liver inflammation, and obesity-related phenotypes. The figure is original and schematic.

Parallel changes in anthropometric, lipid, glycemic, hormonal, inflammatory, and autophagy-related markers suggest potential coordinated host–microbial metabolic adaptations. However, most available evidence derives from observational designs with limited adjustment for confounding variables such as dietary composition and body weight change. Although causality cannot be established, the convergence of microbiome alterations and systemic metabolic improvements supports the hypothesis that RF acts through integrated gut–organ axis mechanisms rather than isolated physiological pathways (Figure 3).

Variations in geographic location, cultural dietary practices, fasting duration, and sequencing platforms (e.g., 16S rRNA vs. metagenomics) may substantially influence reported outcomes, thereby limiting direct comparability between studies. It is important to note that most human studies discussed above are observational in design, and reported associations between microbiome composition, metabolic markers, and anthropometric variables were often derived from correlation analyses. In several studies, adjustment for potential confounding factors such as age, sex, baseline metabolic status, dietary intake, medication use, and particularly body weight change was either limited or not consistently reported. Given that body weight reduction itself can independently influence gut microbiota composition and metabolic outcomes, the extent to which observed associations are mediated directly by fasting versus secondary to weight loss remains to be fully clarified. Future controlled trials incorporating multivariable adjustment models are needed to disentangle these effects.

## 6 Perspective and future directions

RF represents a culturally embedded model of TRE that offers valuable insights into the dynamic interplay between meal timing, gut microbial ecology, and host metabolism. While current evidence suggests that RF may be associated with beneficial modulation of gut microbial diversity and metabolism, further large-scale, multiethnic cohort studies are needed to validate these findings across different populations and dietary habits. It is important to note that baseline metabolic status, medication use, dietary composition, and cultural or geographic variability may influence individual responses to RF, representing potential confounders that should be considered in interpreting these findings. Longitudinal designs assessing post-Ramadan microbiome stability and the sustainability of metabolic improvements would clarify the durability of these effects. Additionally, integrative multi-omics approaches, combining metagenomics, metabolomics, and host transcriptomics, could elucidate the functional consequences of fasting-induced microbial shifts and identify key microbial metabolites mediating health benefits (9, 78, 91).

Mechanistic links between RF, SCFA production, bile acid metabolism, and barrier integrity remain underexplored. Emerging data highlight specific taxa such as *A. muciniphila* and *F. prausnitzii* as key players in maintaining mucosal homeostasis and metabolic regulation during fasting. Future investigations should dissect how insights derived from RF relate to microbial enzymatic activities involved in bile salt hydrolase function, which are critical regulators of host glucose and lipid metabolism via FXR and TGR5 pathways (77, 92, 93).

From a clinical perspective, these findings support the need for controlled trials using TRE protocols to evaluate cardiometabolic outcomes, including whether alignment of the eating window during daytime versus nighttime leads to distinct microbiome and metabolic effects. Interindividual variability may be influenced by genetics, baseline microbiota, and lifestyle factors. Moreover, exploration of RF’s impact on extraintestinal health domains, including neurocognitive function and immune regulation, could unveil broader systemic effects mediated by gut microbiota (2, 81).

Technological advances in sequencing and bioinformatics now enable high-resolution analysis of gut microbial communities and their metabolic capacities. Future research should prioritize controlled intervention studies with rigorous dietary monitoring, standardized microbiome sampling, and incorporation of circadian biology markers. These approaches may help clarify causal relationships and temporal dynamics between fasting, microbiota, and host metabolic pathways. Additionally, experimental models may elucidate specific microbial contributions to RF-mediated metabolic outcomes and inflammation modulation (3, 94).

Finally, considering the global rise in metabolic disorders, *RF*, as a culturally practiced lifestyle pattern, offers valuable observational insights with potential relevance for public health research. A deeper understanding gained through such research could, in turn, inform the development of culturally sensitive health education and awareness programs that respect the religious significance of RF while promoting community health. Expanding access to microbiome-guided personalized fasting protocols could enhance adherence and maximize benefits. Additionally, exploring combinations of RF with other lifestyle modifications, including physical activity and dietary quality improvement, may yield synergistic effects on gut microbiota and metabolic health. Altogether, this review highlights RF as a potentially valuable ecological model to deepen our understanding of time-restricted eating, microbiome-host interactions, and metabolic regulation. Addressing current gaps through multidisciplinary and longitudinal research will pave the way for more targeted, evidence-based public health recommendations and personalized nutritional interventions. Importantly, RF is a religious practice with cultural and spiritual significance, not a clinical intervention. Unlike TRE protocols, which can be implemented experimentally or therapeutically across diverse populations, RF is intrinsically tied to the month of Ramadan and the Islamic faith. Therefore, while RF provides a valuable ecological model for studying the effects of time-restricted feeding under real-world conditions, its health insights should inform research on TRE and other fasting patterns that are adaptable for broader clinical and public health applications. Recognizing this distinction ensures both scientific integrity and cultural respect in translating findings from observational studies of religious practices into actionable health strategies (Table 2).

**Table 2:** The role of RF in modulating microbiome and metabolic pathways.

Author	Year	Study design	Population (n)	Control/Comparator	Time points	Microbiome outcomes	Metabolic/Biochemical outcomes	Brief study-level limitations	References
Studies assessing gut microbiome									
Su et al.	2021	Prospective observational	Healthy adults (*n* = 30)	Pre- vs. post-Ramadan	Before, end, post-Ramadan follow-up	Increased Diversity; increased *Lachnospiraceae*, *Ruminococcaceae*; reversible post-fasting	Decreased Body weight; decreased TG; slight decreased glucose; stable liver enzymes	Observational design; limited confounder adjustment; modest sample size	(76)
Sakandar et al.	2025	Prospective cohort (RF)	Adults observing RF (*n* = 6)	Baseline vs. during/after RF	Multiple time points	Increased Alpha diversity; taxa shifts incl. *Clostridium perfringens, Coprococcus*	Altered AA, carbohydrate, vitamin pathways; SCFA variability	Very small sample size; limited generalizability; exploratory findings	(82)
Özkul et al.	2019	Prospective observational	Healthy adults (*n* = 9)	Pre- vs. post-Ramadan	Before and end Ramadan	Increased *Akkermansia muciniphila*; increased *Bacteroides fragilis* group	Decreased Fasting glucose; decreased total cholesterol	Small cohort; no dietary control; short duration	(77)
Saglam et al.	2023	Observational cohort	Healthy adults (*n* = 12)	Pre- vs. post-Ramadan	Before and end Ramadan	Decreased *Firmicutes*; increased *Proteobacteria*; decreased *Blautia*, *Coprococcus*; increased *Escherichia/Shigella*	Correlations with diet (protein, vegetables, nuts)	Observational correlations; unclear control for diet/energy intake	(78)
Ali et al.	2021	Multicenter observational	Chinese & Pakistani adults (*n* = 34)	Pre- vs. post-Ramadan	Before and end Ramadan	Ethnicity-specific genus shifts	Dietary fat/sweets correlated with genera	Population heterogeneity; dietary variability; no causal inference	(79)
Jo et al.	2023	Prospective observational	Healthy adults (*n* = 20)	Pre- vs. post-Ramadan	Before and after Ramadan	Decreased SCFAs; diversity changes; *Prevotella*, *Blautia* dominant	Functional gene profile differences	Inconsistent SCFA findings; limited longitudinal follow-up	(80)
Özkul et al.	2020	Prospective observational	Healthy adults (*n* = 9)	Pre- vs. post-Ramadan	Before and end Ramadan	Increased Richness; increased *Butyricicoccus, Faecalibacterium, Roseburia*	Not assessed	Small sample; lack of metabolic data	(9)
Selen et al.	2024	Prospective cohort	Overweight/obese adults (*n* = 10)	Pre- vs. post-Ramadan	Before and end Ramadan	Increased Alpha/beta diversity; decreased F/B ratio	Decreased Weight; increased HDL-C; decreased TG	Small sample; weight loss not controlled as confounder	(81)
Studies assessing metabolic/hormonal outcomes without microbiome analysis									
Samaan et al.	2023	Prospective cohort	Before and end Ramadan	Hemodialysis patients (*n* = 45)	Pre- vs. post-Ramadan	Decreased hsCRP, TMAO, PLR, NLR	No	Clinical population; no microbiome data; limited generalizability	(84)
Fakhrzadeh et al.	2003	Observational	Before and end Ramadan	Healthy adults (*n* = 91)	Pre- vs. post-Ramadan	Decreased Waist circumference; decreased LDL-C	No	Observational design; lack of dietary/lifestyle control	(87)
Madkour et al.	2023	Prospective metabolomics study	Baseline and end Ramadan	Healthy adults (*n* = 25)	Pre- vs. post-fasting	23 metabolites decreased; 4 increased; lipid & weight improvements	No	No microbiome assessment; small sample size; short-term analysis	(83)
Dastghaib et al.	2025	Prospective cohort	Baseline and end	Healthy adults (*n* = 50)	Pre- vs. post-30 days fasting	Increased autophagy markers; no adverse biochemical changes	No	No microbiome data; limited mechanistic linkage to gut pathways	(86)
Hassanein et al.	2021	Prospective cohort	Multiple	Low–moderate risk T2DM (*n* = 342)	During vs. pre/post Ramadan	Modest weight decreased; stable glycemic control	No	Observational design; no microbiome data; potential medication confounding	(85)
Mustafa et al.	2024	Prospective cohort	Before and end Ramadan	Healthy adults (*n* = 30)	Pre- vs. post-Ramadan	Decreased insulin, leptin; increased Ghrelin; improved lipid profile	No	Small sample size; no microbiome data; limited confounder adjustment	(88)
Al Zunaidy et al.	2024	Prospective cohort	Before and end Ramadan	Pre/post-menopausal women (*n* = 62)	Pre- vs. post-Ramadan	Decreased Weight, TG; increased HDL, antioxidant markers	No	Population-specific (female only); no microbiome assessment	(89)
Abdelfattah et al.	2025	Prospective cohort	Before and end Ramadan	Athletes (*n* = 15)	Pre- vs. post-Ramadan	Decreased Weight, inflammatory markers; improved lipids	No	Small, specialized population; no microbiome data; limited generalizability	(90)

### 6.1 Limitations of current evidence

Limitations of current evidence translating insights derived from RF into clinically applicable TRE–based interventions for metabolic disease prevention and management. Despite growing interest in RF as a model of time-restricted eating, several important limitations characterize the current body of evidence. First, most available studies are observational and lack randomized controlled designs, limiting causal inference. Sample sizes are generally small, and many investigations are conducted within single geographic or ethnic populations, reducing generalizability. Second, dietary intake during Ramadan is highly heterogeneous and often not rigorously quantified. Variability in macronutrient composition, fiber intake, hydration status, sleep patterns, and physical activity may independently influence gut microbiota composition and metabolic outcomes, making it difficult to isolate the specific effects of fasting duration or feeding rhythm. Third, microbiome analyses across studies employ differing sequencing platforms, bioinformatic pipelines, and taxonomic resolution, contributing to inconsistencies in reported microbial shifts. Few studies incorporate functional metagenomics, metabolomics, or longitudinal follow-up beyond the fasting period. Fourth, many studies do not adequately control for weight loss, which itself can independently alter microbial composition and metabolic markers. Thus, distinguishing fasting-specific effects from energy-restriction–related changes remains challenging. Collectively, these limitations underscore the need for standardized, well-controlled, longitudinal human studies integrating dietary assessment, multi-omics approaches, and mechanistic biomarkers. Therefore, the observed associations should not be interpreted as evidence of causality.

## 7 Conclusion

This review demonstrates that RF robustly remodels the gut microbiota, fostering greater diversity and enriching SCFA-producing, barrier-protective taxa that underlie metabolic benefits such as weight loss, improved glycemic control, and enhanced lipid profiles. By mimicking TRE without caloric restriction, RF reveals how structured feeding–fasting cycles counteract dysbiosis-driven pathologies, including endotoxemia, bile acid dysregulation, and inflammation. Human studies consistently show transient yet clinically meaningful microbial and metabolic shifts, while previous animal research highlights the circadian and mechanistic foundations of these effects. Although the post-fasting reversibility of changes underscores the importance of sustained adherence, the cultural ubiquity of RF positions it as a valuable observational model for studying strategies to combat rising metabolic disorders. Rigorous, large-scale interventions integrating multi-omics approaches are needed to clarify causal pathways and support personalized applications, ultimately helping translate microbiome science into preventive medicine. Given the predominantly observational nature of the available studies, these findings should be interpreted as associative rather than causal.
